# Highly Sensitive SPAD-Based Receiver for Dimming Control in LiFi Networks †

**DOI:** 10.3390/s23104673

**Published:** 2023-05-11

**Authors:** Mohamad Hijazi, Shenjie Huang, Majid Safari

**Affiliations:** School of Engineering, The University of Edinburgh, Edinburgh EH9 3FD, UK; shenjie.huang@ed.ac.uk (S.H.); majid.safari@ed.ac.uk (M.S.)

**Keywords:** visible light communication (VLC), orthogonal frequency division multiplexing (OFDM), light fidelity, dimming control, single-photon avalanche diode (SPAD)

## Abstract

Visible light communication (VLC) is an emerging mode of wireless communication that supports both illumination and communication. One essential function of VLC systems is the dimming control, which requires a sensitive receiver for low-light conditions. The use of an array of single-photon avalanche diodes (SPADs) is one promising approach to enhancing receivers’ sensitivity in a VLC system. However, because of the non-linear effects brought on by the SPAD dead time, an increase in the brightness of the light might degrade its performance. In this paper, an adaptive SPAD receiver is proposed for VLC systems to ensure reliable operation under various dimming levels. In the proposed receiver, a variable optical attenuator (VOA) is used to adaptively control the SPAD’s incident photon rate according to the instantaneous received optical power so that SPAD operates in its optimal conditions. The application of the proposed receiver in systems with various modulation schemes is investigated. When binary on–off keying (OOK) modulation is employed due to its good power efficiency, two dimming control methods of the IEEE 802.15.7 standard based on analogue and digital dimming are considered. We also investigate the application of the proposed receiver in the spectral efficient VLC systems with multi-carrier modulation schemes, i.e., direct current (DCO) and asymmetrically clipped optical (ACO) orthogonal frequency division multiplexing (OFDM). Through extensive numerical results, it is demonstrated that the suggested adaptive receiver outperforms the conventional PIN PD and SPAD array receivers in terms of bit error rate (BER) and achievable data rate.

## 1. Introduction

The exponential growth in data capacity and demand for transmission bandwidth has worsened the scarcity of the radio-frequency (RF) spectrum, making it increasingly challenging to provide access to transmission bandwidth in wireless communication networks, particularly in the fifth generation (5G) and upcoming sixth generation (6G) [1]. To overcome this dilemma, a possible solution has been proposed by shifting towards the terahertz (THz) range, around 10 THz [2]. However, this approach has its own set of difficulties, including power demands, device complexity, and the cost of components such as sources, power amplifiers, antennas, and subharmonic mixers. Alternatively, the ongoing strain on RF-based wireless technologies could be reduced by using the free visible spectrum (380–780 THz) to provide visible light communications (VLCs) using energy-efficient light-emitting diode (LED)-based lighting fixtures in indoor and potential laser diodes (LDs) to outdoor environments [1]. In fact, compared to RF systems, visible and infrared wireless communications may be the most practical technology for illumination, data communications, and localization in most indoor environments because they are low in cost, have high security, and are immune to electromagnetic interference [3].

Future Internet of Things (IoT) applications will have a wide range of performance requirements, such as high data rates and dependable time-sensitive networking. This has led to an increased interest in alternative networking technologies. One such technology is indoor light fidelity (LiFi), which uses light waves to transmit data instead of radio waves [4]. In recent years, researchers have made significant progress in developing LiFi technology that can provide high data rates and low latency. However, one of the key challenges in LiFi networks is maintaining consistent high network performance regardless of lighting level. To address this challenge, researchers have proposed various techniques, such as adaptive modulation and coding schemes, to ensure that LiFi networks can operate reliably under different lighting conditions. In addition, researchers have also explored the potential applications of LiFi technology. For example, LiFi can be used for indoor localization, where the location of a device can be determined based on the strength of the received light signal, in electromagnetic-sensitive industries, or in crowded spaces filled with a lot of RF communication waves [5,6,7,8]. Other studies have investigated the potential of deep learning-based LiFi systems to address challenges and maintain high network performance in indoor environments with varying geometrical configurations and user behavior, including device orientation and blockage. Results have shown that these systems offer superior performance compared to conventional channel estimation techniques [9].

Introducing dimming capabilities into a VLC/LiFi system will result in significant energy savings and offer end users complete control over the light output, making the entire system suitable for commercial usage. However, dimming support was identified as one of the key challenges in VLC by the IEEE 802.15.7 task group as dimming may affect communication performance to some extent. One efficient method of enhancing the performance of optical wireless communication (OWC) devices under weak power reception due to the dimming control is by utilizing highly sensitive photon-counting receivers, such as a single-photon avalanche diode (SPAD).

A SPAD is an avalanche photodiode (APD) that is biased beyond the “Geiger” region’s reverse breakdown voltage. For each detected photon, a SPAD launches billions of electron–hole pair creations that result in a big current detection, and so may be described as a single photon counter. Poisson statistics can be used to represent the photon detection process of an ideal photon counter or in low photon arrival rate regimes [10]. Non-ideal effects, including dead time, photon-detection efficiency (PDE), dark count rate (DCR), afterpulsing, and crosstalk, affect the performance of practical SPAD-based receivers. Dead time is the period during which the SPAD is insensitive to incoming photons and unable to detect any subsequent events. In particular, because of the nonlinearity induced by dead time, SPAD receivers’ observed photon counts deviate significantly from the Poisson distribution [11]. As a way to reduce the dead time effect and hence increase photon-counting capabilities, a SPAD array is widely used that outputs the superposition of the photon counts of each individual SPAD, and the overall photon counts can be further approximated as Gaussian [12].

SPADs have become increasingly popular in recent years due to their high sensitivity and low noise characteristics, making them ideal for use as optical receivers in various applications such as quantum key distribution [13], LiDAR [14], and time-of-flight (ToF) sensing [15]. SPADs are characterized by several key performance metrics including PDE, array size, dead time, afterpulsing, DCR, and crosstalk. For instance, [16] reported a detection efficiency of 30% and a timing jitter of 60 ps for a 25-μm-diameter SPAD at 1550 nm. A dead time compensation model has been presented in [17] by considering reset generated off-pixel which also showed significant PDE improvement. The authors of [18] presented a cost-effective 0.35-μm SPAD in the visible wavelength range, aimed at speeding up the sensing of detector ignition (reduced down to a few hundred picoseconds) which reduces afterpulsing through active quenching of the avalanche current buildup. Furthermore, a recent study [19] demonstrated a high photon-detection efficiency of >19% for a 10-μm-diameter SPAD at 850 nm. Other studies reported on cross-talk characterization of high-fill-factor SPAD arrays and showed that the average cross-talk probability is well below 1% for the shallow-junction SPAD structure with 15.6-μm pitch and 39.9% fill factor [20]. These findings suggest that SPADs have the potential to be employed as photon-counting receivers with high detection efficiency and low noise levels, making them suitable for various applications in photonics.

In this work, we investigate the performance of a SPAD-based VLC system that supports dimming control. In particular, we focus on the bit error rate (BER), data rate, and sensitivity performance. Note that the sensitivity is usually quantified by the minimum detectable signal (MDS) power, which is the minimum optical power required to achieve a specified BER. For a SPAD-based optical receiver, the MDS power can be calculated based on the device’s noise characteristics and the system’s operating parameters.

### 1.1. Related Works

Dimming is an essential feature in VLC systems, allowing for dynamic and energy-efficient illumination control. Over the years, several dimming techniques have been proposed to achieve different illumination levels in VLC systems.

One of the most common techniques is dimming VLC with single-carrier modulation, which involves using pulse-amplitude modulation (PAM) or pulse-width modulation (PWM) to vary the light intensity. Analogue dimming is a continuous dimming method that relies on varying the amplitude of the carrier signal as the case in on–off keying (OOK) transmission, whereas digital dimming is a discrete method that uses PWM to turn the light on and off at a specific duty cycle such as variable OOK (VOOK) transmission. These techniques have been shown to work well in indoor VLC systems [21,22], but they suffer from poor spectral efficiency (SE) when extreme light requirements are targeted.

To overcome these limitations, researchers have explored dimming VLC with multi-carrier modulation, such as optical orthogonal frequency division multiplexing (O-OFDM) with quadrature amplitude modulation (QAM). These techniques use multiple subcarriers to modulate the light intensity, enabling more precise and flexible dimming control. In particular, OFDM-based dimming techniques have been shown to provide higher SE and robustness to inter-symbol interference (ISI) and multipath fading. OFDM and its variations, such as (DC)-biased optical OFDM (DCO-OFDM) [23], asymmetrically clipped optical OFDM (ACO-OFDM) [24], and PAM-DMT [25], have received the most research attention. However, their energy efficiency and SE vary. Hybrid and layered schemes have been proposed to improve SE, including dimming control strategies such as adjusting the DC bias in DCO-OFDM and using reverse-polarity optical OFDM with pulse-width modulation (PWM) during “On” and “Off” stages [26,27,28].

However, most of the previous works on dimming VLC have considered using linear photodetectors (PDs) for signal detection, which have limited sensitivity and dynamic range, strongly limiting the communication performance when the dimming level is low. To address this issue, SPAD receivers can be employed to improve receiver sensitivity and detection accuracy in low-light environments. The application of SPADs in OWC has been widely investigated in the literature, with previous studies demonstrating their ability to achieve high sensitivity, low noise, and excellent timing resolution. For instance, SPADs have been used in OWC systems to improve the performance of underwater communication [29], long-distance free-space communication [30], and high-speed indoor communication [31]. However, the investigation of SPADs’ application in dimming VLC remains scarce, and there is a need for further exploration of their potential in this context to the best of the authors’ knowledge. This work aims to address this research gap by investigating the performance of a novel adaptive SPAD-based receiver in indoor dimmable VLC systems.

### 1.2. Contributions

Motivated by a dual-mode hybrid receiver [32]:

- We propose a novel adaptive receiver employing a SPAD array with a VOA to improve the overall performance of indoor VLC links and satisfy a wide range of dimming requirements.
- The analytical expressions of the signal-to-noise ratio (SNR), BER, and achievable data rate of the VLC dimming systems with the proposed receiver when various modulation schemes are employed are derived. The considered modulation schemes include the single-carrier schemes OOK and VOOK, and also the multi-carrier schemes DCO- and ACO-OFDM.
- We show that in low dimming levels, the proposed receiver benefits from the SPAD’s high sensitivity, but as light brightness increases, the receiver utilizes the VOA to avoid significant non-linear distortion in the SPAD array and maintains superior performance.
- The extensive numerical results demonstrate that the proposed adaptive receiver outperforms both PIN PD and traditional PIN PD and SPAD receivers in terms of BER and achievable data rate under various dimming levels and a wide range of channel conditions.

## 2. System Model

**Figure 1:**
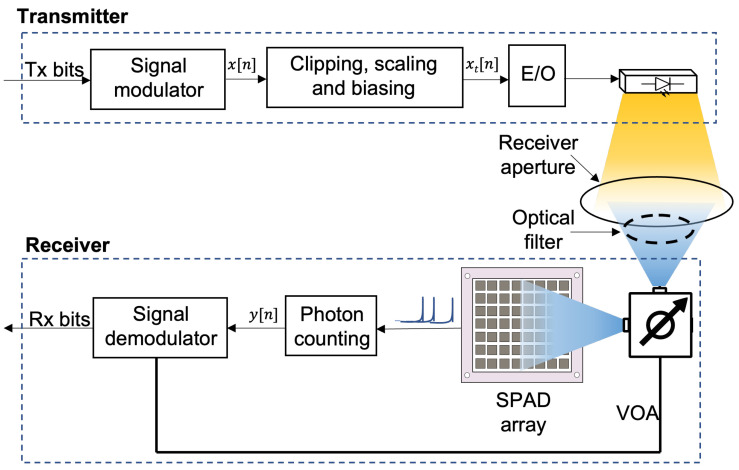
Block diagram of the considered VLC system with the proposed SPAD receiver.

The system model in this study is depicted in Figure 1. Assuming an indoor setting, an array of LEDs is used as a transmitter to generate the modulated, dimmed optical signal, with ambient light also being received from natural and/or unnatural light sources after optical filtering. The signal modulator block denotes one of the various modulation techniques used in this study, which include OOK, VOOK, DCO-OFDM, and ACO-OFDM.

In the considered indoor VLC system, the received optical signal consists of a line of sight (LOS) and/or non-line of sight (NLOS) components and both contribute to the channel pathloss, ζ. Denoting the dimming factor as γ and the maximum average transmitted optical power as ${\bar{P}}_{\mathrm{TX},\max}$, the relationship between γ and the average transmitted optical power ${\bar{P}}_{\mathrm{TX}}$, can be defined as follows

(1) $$\gamma \left(\%\right)=\frac{{\bar{P}}_{\mathrm{TX}}}{{\bar{P}}_{\mathrm{TX},\max}}\times 100\%.$$

To achieve a specific illumination level for dimming purpose, the simplest, most intuitive, and a cost-effective solution is using analogue dimming or continuous current reduction (CCR) [33]. In such a scheme, the dimming control is realized by adjusting the average transmitted light power so that ${\bar{P}}_{\mathrm{TX}}=\gamma {\bar{P}}_{\mathrm{TX},\max}$ holds.

It is worth noting that for OFDM modulation the AC power of the signal will be adjusted accordingly with the change in the dimming level to avoid excessive waveform distortion. Digital dimming, on the other hand, occurs when the signal is digitally altered such as changing its duty cycle, as explained in the next section. With dimming control, the average received optical power, $P_{R}$, can be expressed as

(2) $$P_{R}=\zeta \gamma {\bar{P}}_{\mathrm{TX},\max}.$$

Note that in this work it is assumed that the signaling bandwidth is smaller than the light source’s bandwidth, hence the channel-frequency response is relatively flat across the signaling spectrum and the effect of channel-induced ISI is negligible [34,35].

The SPAD array detector is employed at the receiver of the considered system. After a photon detection, a SPAD has to be quenched for a brief period of time after each avalanche, during which it becomes blind to any incident photon arrivals. This period is referred to as the *“dead time”*. Because of the nonlinearity caused by the dead time, the throughput of OWC systems with SPAD receivers is severely constrained. Typical SPAD receivers use active-quenched (AQ) or passive-quenched (PQ) circuits. PQ SPAD is identified as a paralyzable detector where any counts occurring during the dead time (including signal, dark count, and afterpulse) are not counted but they extend the dead time. Compared with PQ SPAD, the configuration of AQ SPAD is more complex and requires more area and power, but when any photon arrives during the dead time, it is not registered and does not prolong the dead time. In contrast to AQ SPAD, our receiver uses a PQ SPAD array with a large number of micro-cells, which is more sensitive, economical, and has a simpler circuit architecture [36]. The photon transfer function of PQ SPAD is given by [37]

(3) $$\lambda_{D}=\lambda \exp\left(-\lambda \tau_{d}\right),$$

where λ is the received photon rate, $\tau_{d}$ is the dead time, and $\lambda_{D}$ is the detected photon rate. From (3), we can deduce that the detected photon count first increases and subsequently declines with an increase in the incident photon rate. This suggests that the PQ SPAD’s paralysis property results in a nonlinear distortion of the incoming signal. The received photon rate that corresponds to the highest detected photon count is $N_{a}/\tau_{d}$, and the corresponding detected photon count is $N_{a}T_{s}/\left(e\tau_{d}\right)$, where $N_{a}$ is the number of SPAD array pixels, $T_{s}$ is the symbol time, and *e* is Euler’s number [37].

The SPAD array receiver’s relatively restricted dynamic range (defined by the number of micro-cells and the dead time) causes saturation when the received optical power exceeds a certain threshold. As the optical power is increased further, the PQ SPAD detector’s performance degrades significantly. To address this problem, the proposed receiver employs a VOA to attenuate incident light on the SPAD array as necessary. To ensure that the SPAD array receiver performs optimally, the proposed adaptive receiver first determines the optimal transmittance, ξ, from a lookup table and/or the provided equations based on the estimated received signal and ambient power values, $P_{R}$ and $P_{b}$, and then controls the VOA adaptively by the receiver controller that is employed at the signal demodulator unit. The receiver controller monitors both the received signal and background optical power. When the channel condition changes (e.g., a shift in receiver location or orientation or a change in brightness), the controller needs to readjust the optimal transmittance of the VOA to achieve reliable performance. The incident photon rate can be presented as [38]

(4) $$\lambda_{i}\left(\xi \right)=I_{s}\left(\xi \right)\gamma x_{t}+I_{n}\left(\xi \right),$$

where $I_{s}\left(\xi \right)\gamma x_{t}$ and $I_{n}\left(\xi \right)$ denote the signal photon rate and photon rate induced by DCR and background light, respectively. They can be written as

(5) $$\begin{array}{cc} I_{s}\left(\xi \right) & =\frac{\xi \Upsilon_{\mathrm{PDE}}\zeta \left(1+\varrho_{\mathrm{AP}}+\varrho_{\mathrm{CT}}\right)}{E_{\mathrm{ph}}},\\ I_{n}\left(\xi \right) & =\left(\vartheta_{\mathrm{DCR}}+\xi \vartheta_{B}\right)\left(1+\varrho_{\mathrm{AP}}+\varrho_{\mathrm{CT}}\right), \end{array}$$

knowing that $\Upsilon_{\mathrm{PDE}}$ is the PDE of the SPAD, $E_{\mathrm{ph}}$ is the photon energy, $\vartheta_{B}$ represents the background photon rate, and $\vartheta_{\mathrm{DCR}}$, $\varrho_{\mathrm{AP}}$m and $\varrho_{\mathrm{CT}}$ denote the DCR of the array, the afterpulsing and crosstalk probabilities, respectively. It is noted that when ξ=1, the proposed adaptive SPAD receiver converges to a traditional one in the absence of VOA. The background photon rate $\vartheta_{B}$ is equal to $\Upsilon_{\mathrm{PDE}}P_{b}/E_{\mathrm{ph}}$ where $P_{b}$ denotes the received background light power.

For an array of PQ SPADs with $N_{a}$ SPAD pixels and each with a $\tau_{d}$ dead time, the average detected photon count during the time-domain OFDM sample duration, $T_{s}$, can be expressed as [32,37]

(6) $$\mu_{a}\left(x\right)=\lambda_{i}\left(\xi \right)T_{s}\exp\left(-\frac{\lambda_{i}\left(\xi \right)\tau_{d}}{N_{a}}\right).$$

Assuming the sample duration is larger than the dead time, i.e., $T_{s}\geq \tau_{d}$, the variance of the detected photon count of the array is given by [39,40]

(7) $$\begin{array}{c} \sigma_{a}^{2}\left(x\right)=\lambda_{i}\left(\xi \right)T_{s}\exp\left(-\frac{\lambda_{i}\left(\xi \right)\tau_{d}}{N_{a}}\right)-\frac{\lambda_{i}^{2}\left(\xi \right)T_{s}\tau_{d}}{N_{a}}\exp\left(-\frac{2\lambda_{i}\left(\xi \right)\tau_{d}}{N_{a}}\right)\left(2-\frac{\tau_{d}}{T_{s}}\right). \end{array}$$

VLCs employ intensity modulation and direct detection (IM/DD), wherein a direct-detection receiver generates a photocurrent proportionate to the incident optical power. In contrast to standard VLC systems employing linear PDs, which assume a simple additive white Gaussian noise (AWGN) channel, the dead time of SPAD introduces non-linear signal distortion and complicated signal-dependent shot noise, which must be accounted for in the performance analysis [11].

## 3. Single-Carrier Transmission Schemes with Dimming Control

### 3.1. On-Off Keying (OOK)—Analogue Dimming

VLC commonly utilizes OOK modulation, a widely used method where “ON” and “OFF” pulses represent binary bits “1” and “0”, respectively. This method adjusts the brightness of an LED by modulating the forward current through the LED, allowing for dimming by reducing the current. In the case of equiprobable input conditions, the OOK signal transmitted can be expressed as

(8) $$x\left(t\right)=2\gamma {\bar{P}}_{\mathrm{TX},\max}d\;\mathrm{rect}\left(\frac{t}{T_{s}}\right),$$

where d∈{0,1} is the information bit, rect(.) is defined as one for 0<x≤1 and zero otherwise, and $T_{s}$ is the symbol time.

#### 3.1.1. PD Receivers

The BER of the PIN PD detector of OOK scheme can be expressed as [41]

(9) $${\mathrm{BER}}_{\mathrm{PIN}}^{\mathrm{ook}}=Q\left(\sqrt{\frac{\zeta^{2}{ℜ}^{2}\gamma^{2}{\bar{P}}_{\mathrm{TX}}^{2}}{\frac{2KT^{0}R_{b}}{R_{L}}}}\right),$$

where *ℜ* refers to the responsivity of the photodiode, $R_{b}$ denotes the bit rate, subject to the condition $R_{b}\leq B_{\max}$, where $B_{\max}$ represents the maximum electrical bandwidth that can be achieved by LED sources at the transmitter. Additionally, $T^{0}$ indicates the absolute photodiode temperature, and $R_{L}$ is the load resistor for the PIN receiver. It is noted that the effects of shot noise are disregarded, as thermal noise is the primary noise factor for the PIN PD receiver.

The maximum achievable data rate at target BER, $P_{e}^{\mathrm{th}}$, is another metric that will be used, in this work, to compare different schemes. This metric is denoted by $R_{\max}^{\mathrm{ook}}$ and is limited by the system’s bandwidth $B_{\max}$ as

(10) $$R_{\max}^{\mathrm{ook}}=\min\left[\frac{\zeta^{2}{ℜ}^{2}\gamma^{2}{\bar{P}}_{\mathrm{TX}}^{2}}{\left[\frac{2KT^{0}}{R_{L}}\right]{\left[Q^{-1}\left(P_{e}^{\mathrm{th}}\right)\right]}^{2}},B_{\max}\right],$$

where $Q^{-1}\left(.\right)$ denotes the inverse of *Q* function.

#### 3.1.2. Adaptive SPAD Receivers

Differently from the PD receivers, SPAD receivers are shot noise limited and their performance depends on both signal and background optical power.The transmitted OOK signal is assumed to be with an ideal infinite extinction ratio so that the received power for bit “0” is simply $P_{B}$. Therefore, the received photon rate for each micro-cell of the SPAD array when bit “1” and bit “0” are sent can be written as [32]

(11) $$\begin{array}{cc} \lambda_{1}^{\mathrm{ook}}\left(\xi \right) & =\frac{\xi \Upsilon_{\mathrm{PDE}}\left(2\left(\zeta \gamma {\bar{P}}_{\mathrm{TX}}\right)+P_{B}\right)}{N_{a}E_{\mathrm{ph}}},\\ \lambda_{0}^{\mathrm{ook}}\left(\xi \right) & =\frac{\xi \Upsilon_{\mathrm{PDE}}P_{B}}{N_{a}E_{\mathrm{ph}}}, \end{array}$$

It should be noted that the photon rates in (11) are expressed as functions of the adjustable transmittance, ξ. For the purpose of this study, the dead time is regarded as the primary non-ideal effect of the SPAD receiver, and other effects, such as crosstalk and dark count, are disregarded for simplicity. Utilizing the effective photon rate given in Equation (3), the average photon counts corresponding to bit “1” and bit “0” during the symbol duration $T_{s}$ can be computed using Equation (6) as

(12) $$\begin{array}{cc} \mu_{1}^{\mathrm{ook}}\left(\xi \right) & =N_{a}\lambda_{1}^{\mathrm{ook}}\left(\xi \right)T_{s}e^{-\lambda_{1}^{\mathrm{ook}}\left(\xi \right)\tau_{d}},\\ \mu_{0}^{\mathrm{ook}}\left(\xi \right) & =N_{a}\lambda_{0}^{\mathrm{ook}}\left(\xi \right)T_{s}e^{-\lambda_{0}^{\mathrm{ook}}\left(\xi \right)\tau_{d}}. \end{array}$$

The study in [32] assumes equal mean and variance for the detected photon counts, however, we are considering unequal moments which is a more accurate assumption. Therefore, the variance for bit “1” and bit “0” during the symbol duration $T_{s}$ can be calculated from (7) as

(13) $$\begin{array}{cc} \sigma_{1,\mathrm{ook}}^{2}\left(\xi \right) & =\mu_{1}^{\mathrm{ook}}\left(\xi \right)-\frac{\lambda_{1}^{\mathrm{ook}}{\left(\xi \right)}^{2}T_{s}\tau_{d}}{N_{a}}\exp\left(-\frac{2\lambda_{1}^{\mathrm{ook}}\left(\xi \right)\tau_{d}}{N_{a}}\right)\left(2-\frac{\tau_{d}}{T_{s}}\right),\\ \sigma_{0,\mathrm{ook}}^{2}\left(\xi \right) & =\mu_{0}^{\mathrm{ook}}\left(\xi \right)-\frac{\lambda_{0}^{\mathrm{ook}}{\left(\xi \right)}^{2}T_{s}\tau_{d}}{N_{a}}\exp\left(-\frac{2\lambda_{0}^{\mathrm{ook}}\left(\xi \right)\tau_{d}}{N_{a}}\right)\left(2-\frac{\tau_{d}}{T_{s}}\right). \end{array}$$

Assuming Gaussian distribution for detected photon counts of bit “1” (bit “0”), the received BER can be expressed as [42]

(14) $${\mathrm{BER}}_{\mathrm{SPAD}}^{\mathrm{ook}}=Q\left(\frac{\mu_{1}^{\mathrm{ook}}\left(\xi \right)-\mu_{0}^{\mathrm{ook}}\left(\xi \right)}{\sigma_{1,\mathrm{ook}}\left(\xi \right)+\sigma_{0,\mathrm{ook}}\left(\xi \right)}\right).$$

The analytical expression of the optimal transmittance, ξ, which provides the lowest BER, is mathematically intractable. However, the expression of the approximated optimal transmittance assuming equal mean and variance of the detected photon count is given by Proposition 1 in [32]. Hence, in the simulation, we will focus on using the approximated optimal VOA to calculate the BER given in (14). An approximated maximum achievable data rate of the SPAD array with OOK modulation at the target BER, $P_{e}^{\mathrm{th}}$, can be written as [43]

(15) $$\begin{array}{cc} & R_{\mathrm{SPAD}}^{\mathrm{ook}}=\\ & \min\left[\frac{{\left(\sqrt{N\lambda_{1}^{\mathrm{ook}}\left(\xi \right)e^{-\lambda_{1}^{\mathrm{ook}}\left(\xi \right)\tau_{d}}}-\sqrt{N\lambda_{0}^{\mathrm{ook}}\left(\xi \right)e^{-\lambda_{0}^{\mathrm{ook}}\left(\xi \right)\tau_{d}}}\right)}^{2}}{Q^{-1}{\left(P_{e}^{\mathrm{th}}\right)}^{2}},B_{\max}\right]. \end{array}$$

It is worth noting that when the VOA transmittance of the adaptive receiver is fixed at unity, its performance converges to that of a traditional SPAD receiver in the absence of VOA.

### 3.2. Variable On–Off Keying (VOOK)—Digital Dimming

VOOK is a modulation technique that combines OOK and PWM to achieve a specific dimming level. The LEDs are driven by digitally modulated pulses that result in different dimming levels through changes in the duty cycle [21]. The average transmitted optical power for VOOK must be the same as other modulation techniques, namely ${\bar{P}}_{\mathrm{TX}}$, while the peak power of VOOK pulses remains at 2${\bar{P}}_{\mathrm{TX},\max}$ due to digital dimming. The symbol period for VOOK is divided into *m* bits consisting of data (*d*) bits and filler (0) bits. A desired dimming target is achieved by varying the data duty cycle, $\delta_{d}$, which is the ratio of the time the data pulse is on to the symbol duration, $T_{s}$. The inactive portions of the bits are filled with zeros according to the dimming factor, γ. Table 1 shows VOOK codewords of interest, where γ=0% means that no information can be transmitted by VOOK. For VOOK, the highest transmitted optical power is achieved when γ=100%, resulting in a traditional OOK signal.

#### 3.2.1. PD Receivers

To perform VOOK-based dimming, the signal’s duty cycle, $\delta_{d}$, has to be reduced leading to shorter pulses for lower dimming levels than its analogue counterpart, resulting in reduced spectral efficiency as can be seen from Table 1. For instance, when γ=20%, VOOK requires a bandwidth five times higher than that of OOK to maintain a constant data rate. Hence, the maximum achievable data rate for VOOK is limited by the minimum duty cycle, $\delta_{d}^{\min}$, which is directly associated with the minimum achievable dimming level. Assuming that the matched filter integrates solely over the data duty cycle, $\delta_{d}$, and the receiver exploits the maximum available bandwidth by adapting the matched filter to different dimming levels, the output of the matched filter for bits 1 and 0 is $P_{1}={ℜ}^{2}P_{R}^{2}\delta_{d}T$ and $P_{0}=0$, respectively. In the presence of zero-mean Gaussian noise and binary symbols with equal probabilities, the bit error rate (BER) for the VOOK system employing a PIN PD detector can be computed as follows [43]:

(16) $${\mathrm{BER}}_{\mathrm{PIN}}^{\mathrm{vook}}=Q\left(\sqrt{\frac{\delta_{d}\zeta^{2}{ℜ}^{2}\gamma^{2}{\bar{P}}_{\mathrm{TX}}^{2}}{\frac{8KT^{0}R_{b}}{R_{L}}}}\right).$$

Solving (16) for $R_{b}$ at the target BER, $P_{e}^{\mathrm{th}}$, and noting the data rate limitation imposed by $B_{\max}$ and $\delta_{d}$, VOOK maximum achievable data rate for the PD detector can be written as

(17) $$R_{\max}^{\mathrm{vook}}=\max\left[\frac{\delta_{d}\zeta^{2}{ℜ}^{2}\gamma^{2}{\bar{P}}_{\mathrm{TX}}^{2}}{\left[\frac{8KT^{0}}{R_{L}}\right]{\left[Q^{-1}\left(P_{e}^{\mathrm{th}}\right)\right]}^{2}},\delta_{d}^{\min}B_{\max}\right].$$

#### 3.2.2. Adaptive SPAD Receivers

The received photon rate of each PQ SPAD micro-cell when bit “0” and bit “1” are sent for VOOK, can be expressed as [43]

(18) $$\begin{array}{cc} \lambda_{1}^{\mathrm{vook}}\left(\xi \right) & =\frac{\xi \Upsilon_{\mathrm{PDE}}\left(2\delta_{d}\zeta \gamma {\bar{P}}_{\mathrm{TX}}+P_{b}\right)}{N_{a}E_{\mathrm{ph}}},\\ \lambda_{0}^{\mathrm{vook}}\left(\xi \right) & =\frac{\xi \Upsilon_{\mathrm{PDE}}P_{b}}{N_{a}E_{\mathrm{ph}}}, \end{array}$$

where ξ denotes the transmittance of the employed VOA for VOOK modulation. The average photon counts for bit “0” and bit “1” during the VOOK’s symbol duration $T_{s}$ are given by [43]

(19) $$\begin{array}{cc} \mu_{1}^{\mathrm{vook}}\left(\xi \right) & =N_{a}\lambda_{1}^{\mathrm{vook}}\left(\xi \right)T_{s}e^{-\lambda_{1}^{\mathrm{vook}}\left(\xi \right)\tau_{d}},\\ \mu_{0}^{\mathrm{vook}}\left(\xi \right) & =N_{a}\lambda_{0}^{\mathrm{vook}}\left(\xi \right)T_{s}e^{-\lambda_{0}^{\mathrm{vook}}\left(\xi \right)\tau_{d}}, \end{array}$$

Similar to the OOK case, the variance of the photon counts can be expressed as

(20) $$\begin{array}{cc} \sigma_{1,\mathrm{vook}}^{2}\left(\xi \right) & =\mu_{1}^{\mathrm{vook}}\left(\xi \right)-\frac{\lambda_{1}^{\mathrm{vook}}{\left(\xi \right)}^{2}T_{s}\tau_{d}}{N_{a}}\exp\left(-\frac{2\lambda_{1}^{\mathrm{vook}}\left(\xi \right)\tau_{d}}{N_{a}}\right)\left(2-\frac{\tau_{d}}{T_{s}}\right),\\ \sigma_{0,\mathrm{vook}}^{2}\left(\xi \right) & =\mu_{0}^{\mathrm{vook}}\left(\xi \right)-\frac{\lambda_{0}^{\mathrm{vook}}{\left(\xi \right)}^{2}T_{s}\tau_{d}}{N_{a}}\exp\left(-\frac{2\lambda_{0}^{\mathrm{vook}}\left(\xi \right)\tau_{d}}{N_{a}}\right)\left(2-\frac{\tau_{d}}{T_{s}}\right). \end{array}$$

Therefore, the BER expression for PQ-based SPAD array receiver when VOOK modulation is employed can be expressed as

(21) $${\mathrm{BER}}_{\mathrm{SPAD}}^{\mathrm{vook}}=Q\left[\Gamma (\xi )\right]$$

where $\Gamma \left(\xi \right)=\frac{\mu_{1}^{\mathrm{vook}}\left(\xi \right)-\mu_{0}^{\mathrm{vook}}\left(\xi \right)}{\sigma_{1,\mathrm{vook}}\left(\xi \right)+\sigma_{0,\mathrm{vook}}\left(\xi \right)}$.

In the proposed receiver, the controller adjusts ξ so that the SPAD array can operate at its best. The optimal ξ minimizing ${\mathrm{BER}}_{\mathrm{SPAD}}^{\mathrm{vook}}$ is equivalent to the one maximizing the Γ(ξ). With the assumption of equal mean and variance of the detected photon count [32], the approximated optimal ξ for VOOK can be expressed as

(22) $$\xi =\min\left[\frac{2Nhvy_{\mathrm{root},1}}{\Upsilon_{\mathrm{PDE}}\tau_{d}P_{R}},1\right],$$

where $y_{\mathrm{root},1}$ denotes the single positive root of the non-linear equation

(23) $$\frac{2P_{b}}{P_{R}}y-1=\frac{2y}{\sqrt{\frac{P_{b}}{P_{R}+P_{b}}}e^{y}-1},$$

in the regime $y\in (0,\ln\sqrt{\frac{P_{R}+P_{b}}{P_{b}}})$.

The approximated maximum achievable data rate of the SPAD array for VOOK system at the target BER, $P_{e}^{\mathrm{th}}$, can be expressed as

(24) $$R_{\mathrm{SPAD}}^{\mathrm{vook}}=\min\left[\frac{{\left(\sqrt{\mu_{1}\left(\xi \right)}-\sqrt{\mu_{0}\left(\xi \right)}\right)}^{2}}{Q^{-1}{\left(P_{e}^{\mathrm{th}}\right)}^{2}},\delta_{d}^{\min}B_{\max}\right].$$

It should be indicated that (24) requires $\xi <2Nhv\ln\frac{P_{R}+P_{b}}{P_{b}}/2\tau_{d}\Upsilon_{\mathrm{PDE}}P_{R}$ and the derived transmittance value (22) satisfies this condition.

## 4. Multi-Carrier Transmission Schemes with Dimming Control

In order to improve the data rate, SPAD-based OWC with OFDM, which provides higher spectral efficiency compared to OOK, is investigated in this section. We focus on the two most commonly used OFDM schemes, i.e., DCO-OFDM and ACO-OFDM. Note that similar evaluations can also be carried out for the other before-mentioned OFDM variants. Analogue dimming is only considered for such modulation schemes as a result of the time-domain OFDM samples being continuous which makes the codeword design of digital dimming impossible.

### 4.1. DCO-OFDM

In a DCO-OFDM system, the *M*-quadrature amplitude modulation (QAM) modulator at the transmitter converts the input bit stream into a complex symbol stream, where *M* is the constellation size. The symbol stream is then transformed from serial to parallel (S/P) to create vectors that can be used with the inverse fast Fourier transform (IFFT).We consider a *K*-point fast Fourier transform (FFT) operation, in which the information-carrying subcarriers fill the first half of the OFDM frame while leaving the 0-th and K/2-th subcarriers empty leading to a total $K^{\prime }=K/2-1$ information-carrying subcarriers [44]. Denote the symbol allocated to the *k*th subcarrier as X[k]=1,⋯,K, the second half of the OFDM frame is subjected to Hermitian symmetry of the subcarriers, $X\left[l\right]=X^{*}\left[K-l\right]$, for l=1,2,⋯K/2−1, in order to ensure a real-valued time domain signal at the cost of a 50% reduction in the spectral efficiency compared to conventional radio frequency based OFDM (RF-OFDM). The time domain signal x[n], that represents the *n*-th time-domain OFDM sample emitted from the source, can be obtained after the IFFT as

(25) $$x\left[n\right]=\frac{1}{\sqrt{K}}\sum_{k=0}^{K-1}X\left[k\right]\;e^{j2\pi \frac{kn}{K}}\;;\;0\leq n\leq K-1$$

where $j=\sqrt{\left(-1\right)}$ is the imaginary number. As long as *K* is fairly high (K≥64), the central limit theorem (CLT) states that the amplitude of x[n] is approximately zero-mean Gaussian distributed [35]. Therefore, the variance of X[k] is adjusted to $\sigma_{X}^{2}=K/\left(K-2\right)$ in order to ensure that x[n] is with unit variance while taking into account the uniform power distribution over the subcarriers [34]. Since actual light sources have constrained dynamic ranges [$P_{\min},P_{\max}$] that correspond to the minimum and maximum optical power of the linear dynamic range of the LED, respectively, and the relatively high signals produced, x[n] must be correctly clipped to efficiently utilize that dynamic range.The clipping would ideally be within the range $\left[\kappa_{b},\;\kappa_{t}\right]$ which denote the predefined normalized bottom and top clipping levels, respectively. Therefore, the clipped signal, $\hat{x}\left[n\right]$, can be expressed as

(26) $$\hat{x}\left[n\right]=\left\{\begin{array}{cc} \kappa_{t} & \mathrm{if}\;\;\;x\left[n\right]\geq \kappa_{t},\\ x[n] & \mathrm{if}\;\;\;\kappa_{b}<x\left[n\right]<\kappa_{t},\\ \kappa_{b} & \mathrm{if}\;\;\;x\left[n\right]\leq \kappa_{b}. \end{array}\right.$$

According to the Bussgang theorem [45], the nonlinear distortion effects caused by the clipping operation can be approximated by

(27) $$\hat{x}\left[n\right]=\eta x\left[n\right]+w^{\mathrm{clip}}\left[n\right],$$

where η denotes the clipping attenuation factor and $w^{\mathrm{clip}}\left[n\right]$ is the clipping noise. It is noted that the clipping noise $w^{\mathrm{clip}}\left[n\right]$ is not correlated with the signal x[n], hence, based on the Bussgang theorem, we can get $\eta =E\left[x\left[n\right]\hat{x}\left[n\right]\right]/\sigma_{x}^{2}$ and $E[x\left[n\right]w^{\mathrm{clip}}\left[n\right]]=0$ where $\sigma_{x}^{2}$ is the variance of x[n] which is unity throughout the paper, and E[.] is the expectation operator. Therefore, the moments of the clipping noise can be expressed as

(28) $$E\left[w^{\mathrm{clip}}\left[n\right]\right]=E\left[\hat{x}\left[n\right]\right],\;\sigma_{\mathrm{clip}}^{2}=E\left[{\hat{x}}^{2}\left[n\right]\right]-\eta \sigma_{x}^{2}-E^{2}\left[\hat{x}\left[n\right]\right],$$

To achieve a maximal signal SNR and to transform the electrical signal into an optical one, the linear dynamic range 0 and $P_{\max}$ should be mapped by the normalised clipping levels, $\kappa_{b}$ and $\kappa_{t}$. Hence, to form such mapping, scaling, DC biasing, and digital-to-analogue conversion are required stages before the light source can be driven. In practise, the optical power of the source’s *n*th time-domain OFDM sample is provided by [34]

(29) $$x_{t}\left[n\right]=\alpha \hat{x}\left[n\right]+P_{\mathrm{bias}},$$

where α is the scaling factor and $P_{\mathrm{bias}}$ is the input biasing current which should be carefully chosen to ensure that it is non-negative and fulfils the light source’s unipolarity property. To perform such mapping, the following equations should be satisfied to calculate the scaling factor, α, and $P_{\mathrm{bias}}$

(30) $$\begin{array}{cc} \alpha =\frac{P_{\max}}{\kappa_{t}-\kappa_{b}}, & \;P_{\mathrm{bias}}=\frac{-P_{\max}\kappa_{b}}{\kappa_{t}-\kappa_{b}}. \end{array}$$

The average transmit optical power is defined as $E\left(x_{t}\left[n\right]\right)={\bar{P}}_{\mathrm{TX}}$, and it can be expressed as [34]

(31) $${\bar{P}}_{\mathrm{TX}}=\alpha \left[\phi \left(\kappa_{b}\right)-\phi \left(\kappa_{t}\right)+\kappa_{t}Q\left(\kappa_{t}\right)+\kappa_{b}Q\left(-\kappa_{b}\right)\right]+P_{\mathrm{bias}},$$

where

(32) $$\phi \left(x\right)=\frac{1}{\sqrt{2\pi }}\exp\left(-\frac{x^{2}}{2}\right),$$

is the probability density function (PDF) of a standard Gaussian distribution and Q(.) denotes the Q-function. It is noted that symmetric clipping is employed throughout this paper i.e., $\kappa_{t}=-\kappa_{b}=\kappa$.

#### 4.1.1. PD Receivers

The received discrete time domain signal y[n] is obtained after removing the scaling factor, DC-bias and the CP. The received electrical waveform is given by

(33) $$y\left[n\right]=hx_{t}\left[n\right]+w^{\mathrm{rx}}\left[n\right],$$

where h=γℜζ and $w^{\mathrm{rx}}\left[n\right]$ is the receiver noise with variance $\sigma_{\mathrm{rx}}^{2}$. Applying the FFT property to the received signal y[n] translates the signal to the frequency domain and converts the convolution operation into a multiplication one. In the frequency domain, the clipping noise $w^{\mathrm{rx}}\left[n\right]$ is transformed into additive Gaussian noise due to CLT. Therefore, an additional additive Gaussian noise component with zero-mean and variance $\sigma_{\mathrm{clip},\mathrm{DCO}}^{2}$ is present at each modulated subcarrier. The FFT operation is given by the following:

(34) $$Y\left[k\right]=\frac{1}{\sqrt{K}}\sum_{n=0}^{K-1}y\left[n\right]\;e^{-j2\pi \frac{kn}{K}},\;0\leq k\leq K-1$$

The received signal can be calculated as:

(35) $$\begin{array}{cc} Y[k] & =H\left(\eta X\left[k\right]+W^{\mathrm{clip}}\left[k\right]\right)+W^{\mathrm{rx}}\left[k\right],\\ \; & =\eta HX\left[k\right]+HW^{\mathrm{clip}}\left[k\right]+W^{\mathrm{rx}}\left[k\right], \end{array}$$

where *H*, $W^{\mathrm{clip}}\left[k\right]$ and $W^{\mathrm{rx}}\left[k\right]$ denote the FFT of h[n], $w^{\mathrm{clip}}\left[n\right]$, and $w^{\mathrm{rx}}\left[n\right]$, respectively. It can be seen that the received signal can be written as the summation of the desired signal and two zero mean noises, clipping noise and receiver noise. Therefore, the SNR of the *k*th subcarrier for DCO-OFDM can be expressed as [34]

(36) $$\Phi {\left[k\right]}^{\mathrm{DCO}}=\frac{\eta_{\mathrm{DCO}}^{2}\frac{K}{K-2}{\left|H\right|}^{2}}{\sigma_{\mathrm{clip},}^{2}\;\mathrm{DCO}{\left|H\right|}^{2}+\sigma_{\mathrm{rx}}^{2}},$$

where $\sigma_{\mathrm{rx}}^{2}$ is the receiver noise given by $\sigma_{\mathrm{rx}}^{2}=N_{0}B$, $B=1/T_{s}$ is the signaling bandwidth, $T_{s}$ is the symbol period, and $N_{0}=4\varepsilon T^{0}/R_{L}$ is the thermal noise power spectral density (PSD) with Boltzman constant, ε, load resistor temperature, $T^{0}$, and load resistance, $R_{L}$. The attenuation factor and clipping noise variance are given by [34,46]

(37) $$\eta_{\mathrm{DCO}}=Q\left(\kappa_{b}\right)-Q\left(\kappa_{t}\right),$$

(38) $$\begin{array}{cc} \sigma_{\mathrm{clip},\mathrm{DCO}}^{2}= & \eta_{\mathrm{DCO}}+\kappa_{b}\phi \left(\kappa_{b}\right)-\kappa_{t}\phi \left(\kappa_{t}\right)+\kappa_{b}^{2}\left[1-Q\left(\kappa_{b}\right)\right]+\kappa_{t}^{2}Q\left(\kappa_{t}\right)\\ \; & -\eta_{\mathrm{DCO}}^{2}-{\left[\phi \left(\kappa_{b}\right)-\phi \left(\kappa_{t}\right)+\kappa_{b}\left[1-Q\left(\kappa_{b}\right)\right]+\kappa_{t}Q\left(\kappa_{t}\right)\right]}^{2}, \end{array}$$

#### 4.1.2. Adaptive SPAD Receivers

Theoretical performance analysis of SPAD-based OWC systems with DCO-OFDM and ACO-OFDM was conducted in [47,48], and the derived equations will be used in this study to be suited for dimming scenarios.

It is noted that (7) is a nonlinear function of the signal amplitude causing this shot noise to be signal-dependent and more complicated than the traditional APD shot noise, whose power is proportional to the signal amplitude [49]. Therefore, there are two nonlinear distortions in the SPAD OFDM system under consideration. Similarly to linear photodetectors, the clipping-induced distortion caused by the light source’s low dynamic range as presented in (26) is one component. The second is the special distortion caused by SPAD as introduced in (7). The combined nonlinear distortion of the transmitted signal x[n] can be written as [47]

(39) $$\mu_{a}\left(x\left[n\right]\right)=\left\{\begin{array}{cc} \left(\beta_{1}\kappa_{t}+\beta_{2}\right)\;T_{s}\;\exp\left[-\frac{\left(\beta_{1}\kappa_{t}+\beta_{2}\right)\tau_{d}}{N_{a}}\right], & x\left[n\right]\geq \kappa_{t},\\ \left(\beta_{1}x\left[n\right]+\beta_{2}\right)\;T_{s}\;\exp\left[-\frac{\left(\beta_{1}x\left[n\right]+\beta_{2}\right)\tau_{d}}{N_{a}}\right], & \kappa_{b}<x\left[n\right]<\kappa_{t},\\ \left(\beta_{1}\kappa_{b}+\beta_{2}\right)\;T_{s}\;\exp\left[-\frac{\left(\beta_{1}\kappa_{b}+\beta_{2}\right)\tau_{d}}{N_{a}}\right], & x\left[n\right]\leq \kappa_{b}. \end{array}\right.$$

where

(40) $$\beta_{1}=\alpha I_{s}\left(\xi \right),\;\mathrm{and}\;\beta_{2}=I_{s}\left(\xi \right)P_{\mathrm{bias}}+I_{n}\left(\xi \right).$$

and $I_{s}\left(\xi \right)$ with $I_{n}\left(\xi \right)$ can be calculated from (5). According to (27), the nonlinear distortion in an OFDM-based system can be quantified by a gain factor (η) and an additional distortion-induced noise ($w^{\mathrm{clip}}\left[n\right]=w_{d}\left[n\right]$). The gain factor(η) can be expressed as [47]

(41) $$\begin{array}{cc} \eta_{\mathrm{DCO}}= & \frac{\beta_{1}^{2}\tau_{d}T_{s}}{\sqrt{2\pi }N_{a}}\left\{\exp\left[-\frac{\kappa_{t}^{2}}{2}-\frac{\tau_{d}}{N_{a}}\left(\beta_{1}\kappa_{t}+\beta_{2}\right)\right]-\exp\left[-\frac{\kappa_{b}^{2}}{2}-\frac{\tau_{d}}{N_{a}}\left(\beta_{1}\kappa_{b}+\beta_{2}\right)\right]\right\}\\ \; & +\beta_{1}T_{s}e^{-\frac{\beta_{2}\tau_{d}}{N_{a}}+\frac{\beta_{1}^{2}\tau_{d}^{2}}{2N_{a}^{2}}}\left[1+\frac{\beta_{1}^{2}\tau_{d}^{2}}{N_{a}^{2}}-\frac{\beta_{2}\tau_{d}}{N_{a}}\right]\times \left[Q\left(\kappa_{b}+\frac{\beta_{1}\tau_{d}}{N_{a}}\right)-Q\left(\kappa_{t}+\frac{\beta_{2}\tau_{d}}{N_{a}}\right)\right]. \end{array}$$

The variance of the distortion induced noise, $\sigma_{w_{d}}^{2}$, can be calculated by

(42) $$\sigma_{w_{d}}^{2}=E\left\{\mu_{a}^{2}\left(x\right)\right\}-E^{2}\left\{\mu_{a}\left(x\right)\right\}-\eta_{\mathrm{DCO}}^{2},$$

where $E\{\mu_{a}^{2}\left(x\right)\}$ and $E^{2}\{\mu_{a}\left(x\right)$ can be retrieved from ([49] Equation (33)) and ([49], Equation (37)), respectively.

After serial-to-parallel (S/P) mapping, the detected photon count at the output of the SPAD array during the sample period $T_{s}$ is denoted as y[n]. As the SPAD array size increases, CLT is valid causing y[n] to be approximately Gaussian distributed with mean and both variances given by (6), (7), and (42), respectively. Hence, taking into account (27), y[n] can be represented as

(43) $$y\left[n\right]=\eta_{\mathrm{DCO}}x\left[n\right]+w_{a}\left[n\right]+w_{d}\left[n\right],$$

where $w_{a}\left[n\right]$ depicts signal-dependent shot noise that is Gaussian distributed with zero mean and signal-dependent variance (7), and $w_{d}\left[n\right]$ is the distortion-induced noise according to the Bussgang theorem. The photon-counting signal y[n] is transformed back to the frequency domain using FFT as in (34) and can be expressed as

(44) $$Y\left[k\right]=\eta_{\mathrm{DCO}}X\left[k\right]+W_{a}\left[k\right]+W_{d}\left[k\right],$$

where $W_{a}\left[k\right]$ and $W_{d}\left[k\right]$ denote the FFT of $w_{a}\left[n\right]$ and $w_{d}\left[n\right]$, respectively. Different from the distortion-induced noise where both the time-domain and the frequency-domain noise variances are equal, $\sigma_{W_{d}}^{2}=\sigma_{w_{d}}^{2}$, the shot noise, $w_{a}\left[n\right]$, is signal dependent, and hence its frequency domain noise variance can be calculated as seen in ([49], Equation (46)).

Due to the averaging effect of the FFT operation, the signal-dependent time-domain shot noise becomes signal-independent in the frequency-domain noise. Therefore, both noise terms in the received signal indicated in (44) are uncorrelated with the signal, making the channel model a conventional additive Gaussian noise channel. Note that, in contrast to the original OWC channel model, the equivalent additive Gaussian channel model for OFDM SPAD-based OWC system in (44) is not restricted by the non-negativity condition of the intensity modulation. Hence, the SNR of the received signal can be written as

(45) $$\Gamma_{\mathrm{DCO}}\left(\xi \right)=\frac{\eta_{\mathrm{DCO}}^{2}\frac{K}{K-2}}{\sigma_{W_{d}}^{2}+\sigma_{W_{a}}^{2}},$$

where the term ($\eta_{\mathrm{DCO}}^{2}K)/\left(K-2\right)$ refers to the received electrical signal power with $|\eta_{\mathrm{DCO}}|$ and can be used to measure the dimmed signal.The received symbols can be equalized using a single-tap equalizer. After equalization and parallel-to-serial (P/S) mapping, the received QAM signal can be achieved. The QAM demodulation can then be applied, which results in the recovered bit stream followed by BER calculation.

### 4.2. ACO-OFDM

In ACO-OFDM system, the signal generation is similar to that of DCO-OFDM systems but utilizes a different frame structure. Only the odd subcarriers of the first half of the OFDM frame with index k=1,3,5,⋯,K/2−1 are used to transport the information when performing a *K*-point fast Fourier transform (FFT), whilst the even subcarriers are left unused, leading to a total number of $K^{\prime }=K/4$ information-carrying subcarriers. To get the real-valued symbols following the IFFT operation, Hermitian symmetry is applied to the remaining OFDM frame and a similar time domain signal as (25) is obtained. To ensure that x[n] is with unit variance, the variance of the signal should be $\sigma_{X}^{2}=2$ when taking into account the uniform power distribution over the subcarriers [34]. To guarantee that the time domain signal is transmitted with non-negative values, it has to be properly clipped at a top clipping level, κ. Hence, the clipped signal, $\hat{x}\left[n\right]$, can be expressed as

(46) $$\hat{x}\left[n\right]=\left\{\begin{array}{cc} \kappa & \mathrm{if}\;\;\;x[n]\geq \kappa ,\\ x[n] & \mathrm{if}\;\;\;0<x[n]<\kappa ,\\ 0 & \mathrm{if}\;\;\;x[n]\leq 0. \end{array}\right.$$

Because of the time domain signal structure in ACO-OFDM and DCO-OFDM, the optical power of the *n*th time-domain OFDM sample emitted from the source is given by $x_{t}\left[n\right]=\alpha \hat{x}\left[n\right]$. This will lead to $\alpha =P_{\max}/\kappa$. Therefore, the average transmit optical power is given by

(47) $${\bar{P}}_{\mathrm{TX}}=\alpha \left[1/\sqrt{2\pi }-\phi \left(\kappa \right)+\kappa Q\left(\kappa \right)\right],$$

ACO-OFDM dimming is achieved in a similar manner as that of its DCO-OFDM counterpart, but with a decrease of the dynamic range of the utilized power source, i.e., $\left[0,\gamma P_{\max}\right]$.

#### 4.2.1. PD Receivers

ACO-OFDM’s received signal can be obtained in a similar manner to DCO-OFDM, taking into consideration its different frame structure. Therefore, the SNR of the *k*th subcarrier for ACO-OFDM can be expressed as [34]

(48) $$\Phi {\left[k\right]}^{\mathrm{ACO}}=\frac{2\eta_{\mathrm{ACO}}^{2}{\left|H\left[k\right]\right|}^{2}}{\sigma_{\mathrm{clip}}^{2}{\left|H\left[k\right]\right|}^{2}+\sigma_{\mathrm{rx}}^{2}},$$

The attenuation factor and clipping noise variance are given, respectively, by [34,46]

(49) $$\eta_{\mathrm{ACO}}=\frac{1}{2}-Q\left(\kappa \right),$$

(50) $$\sigma_{\mathrm{clip},\mathrm{ACO}}^{2}=\kappa^{2}Q\left(\kappa \right)-\kappa \phi \left(\kappa \right)+Q\left(\kappa \right)-2Q{\left(\kappa \right)}^{2},$$

#### 4.2.2. Adaptive SPAD Receivers

For this SPAD OFDM system, the combined nonlinear distortion of the transmitted signal x[n] can be derived by substituting (46) and (4) into (6) to obtain

(51) $$\mu_{a}\left(x\left[n\right]\right)=\left\{\begin{array}{cc} \left(\beta_{1}\kappa +\lambda_{n}\right)\;T_{s}\;\exp\left[-\frac{\left(\beta_{1}\kappa +\lambda_{n}\right)\tau_{d}}{N_{a}}\right], & x[n]\geq \kappa ,\\ \left(\beta_{1}x\left[n\right]+\lambda_{n}\right)\;T_{s}\;\exp\left[-\frac{\left(\beta_{1}x\left[n\right]+\lambda_{n}\right)\tau_{d}}{N_{a}}\right], & 0<x[n]<\kappa ,\\ \lambda_{n}\;T_{s}\;\exp\left[-\frac{\lambda_{n}\tau_{d}}{N_{a}}\right], & x[n]\leq 0. \end{array}\right.$$

where $\beta_{1}=I_{s}\left(\xi \right)P_{\max}/\kappa .$

Similar to the DCO-OFDM case, the OFDM signal can be quantified by a gain factor ($\eta_{\mathrm{ACO}}$) and an additional signal-independent noise distortion-induced noise ($w_{d}\left[n\right]$) according to the Bussgang theorem. The gain factor $\eta_{\mathrm{ACO}}$ can be calculated as [48]

(52) $$\begin{array}{cc} \eta_{\mathrm{ACO}}= & \frac{\beta_{1}^{2}\tau_{d}T_{s}}{\sqrt{2\pi }N_{a}}\left\{\exp\left[-\frac{\kappa^{2}}{2}-\frac{\tau_{d}}{N_{a}}\left(\beta_{1}\kappa +\lambda_{n}\right)\right]-\exp\left[-\frac{\tau_{d}\lambda_{n}}{N_{a}}\right]\right\}\\ \; & +\beta_{1}T_{s}e^{-\frac{\lambda_{n}\tau_{d}}{N_{a}}+\frac{\beta_{1}^{2}\tau_{d}^{2}}{2N_{a}^{2}}}\left[1+\frac{\beta_{1}^{2}\tau_{d}^{2}}{N_{a}^{2}}-\frac{\lambda_{n}\tau_{d}}{N_{a}}\right]\times \left[Q\left(\frac{\beta_{1}\tau_{d}}{N_{a}}\right)-Q\left(\kappa +\frac{\beta_{1}\tau_{d}}{N_{a}}\right)\right]. \end{array}$$

The variance of the distortion-induced noise, $\sigma_{w_{d}}^{2}$, can be calculated in a similar manner as that for the DCO-OFDM case from (42). By applying the same principle as in (44), the variance of the shot noise, $W_{a}$, for the considered system with ACO-OFDM, denoted as $\sigma_{W_{a}}^{2}$, is identical to that with DCO-OFDM which has been introduced above in reference [49], Equation (46).

The SNR of the received signal for ACO-OFDM can be written as

(53) $$\Gamma_{\mathrm{ACO}}\left(\xi \right)=\frac{\eta_{\mathrm{ACO}}^{2}\sigma_{X}^{2}}{\sigma_{W_{d}}^{2}+\sigma_{W_{a}}^{2}}.$$

It is noted that the closed-form expression for the BER performance of *M*-QAM in AWGN is presented as a sum of terms in [50]. Using only the first two terms yields an accurate approximation. Therefore, the BER performance of *M*-QAM O-OFDM for the *k*th subcarrier of the considered modulation techniques for both PD and SPAD systems can be written as

(54) $$\begin{array}{c} P_{e}\left[k\right]=\frac{4(\sqrt{M}-1)}{\sqrt{M}\log_{2}\left(M\right)}Q\left(\sqrt{\frac{3\Theta }{M-1}}\right)+\frac{4(\sqrt{M}-2)}{\sqrt{M}\log_{2}\left(M\right)}Q\left(3\sqrt{\frac{3\Theta }{M-1}}\right) \end{array},$$

where Θ is the relevant SNR of the considered OFDM system as in (36), (45), (48) and (53).

In the proposed receiver, the controller should adjust the VOA transmittance, ξ, so that the SPAD array can operate at its best. The optimal ξ minimizing the BER is equivalent to the one maximizing its SNR. Hence, by taking the first derivative of $\Gamma_{\mathrm{DCO}}\left(\xi \right)$ or $\Gamma {(}_{\mathrm{ACO}}\xi )$ with respect to ξ and letting it equal zero, it will lead to a non-linear equation which is mathematically intractable. Therefore, we will find the optimal VOA transmittance, ξ, for the considered O-OFDM systems through an exhaustive search, and a look-up table of the optimal ξ under various $P_{R}$ and $P_{b}$ could be calculated and pre-saved at the receiver. Using the chart, the ideal transmittance of VOA may therefore be chosen for the communication.

To explore the throughput of the SPAD-based OFDM system, the SNR terms of DCO- and ACO-OFDM should be substituted into (54) where we calculate the BER for each subcarrier. Hence, the transmission data rate for the DCO-OFDM system can be written as

(55) $$R_{\max}^{\mathrm{DCO}}=\frac{\log_{2}\left(M\right)\left(K/2-1\right)}{KT_{s}}.$$

As only the odd subcarriers are being transmitted by ACO-OFDM, its throughput will be approximately half of that of DCO-OFDM, i.e., $R_{\max}^{\mathrm{ACO}}=\frac{R_{\max}^{\mathrm{DCO}}}{2}$.

## 5. Numerical Results and Discussion

In this section, BER performance and achievable data rate metrics for the previously discussed modulation techniques are presented. The common parameters used in the simulation are given in Table 2. Because of the differences in their system design and to demonstrate the superiority of our receiver, a relatively small number of SPADs are used for single-carrier systems. Such systems can be implemented in IoT devices that benefit from high power efficiency and do not require high spectral efficiency [51]. For OOK and VOOK modulations, the SPAD detector has $N_{a}=1024$ micro-cells, $\tau_{d}=40$ ns, and $\Upsilon_{\mathrm{PDE}}=0.1$. For the OFDM ones, we aim to explore the application of SPAD receivers in high-speed OWC, hence larger array sizes and less dead time are considered. Therefore, the SPAD detector has $N_{a}=8192$ micro-cells, $\tau_{d}=10$ ns, and $\Upsilon_{\mathrm{PDE}}=0.35$. Because the spectral efficiency of ACO-OFDM is half that of DCO-OFDM, a fair comparison of the schemes should be *M*-QAM ACO-OFDM versus $\sqrt{M}$-QAM DCO-OFDM. Therefore, in the simulation, 16-QAM is used for ACO-OFDM and 4-QAM is considered for DCO-OFDM. We assume that the whole available LED bandwidth $B_{\max}$ is utilized for signal transmission and hence a time-domain symbol duration of $T_{s}=$ 8.35 ns is considered for all modulation schemes. We also consider different ambient light scenarios. Despite using optical filtering, ambient optical power from sources such as sunlight, skylights, and incandescent bulbs can still be a significant factor. To account for this, we have categorized the ambient light power into two levels based on the aperture of our receiver: 1 nW for situations with relatively low ambient light, and 10 nW (approximately 500 lux) for situations where the user is located near a window [52]. It is noted that we assumed that a proper narrowband optical filter with a centre wavelength aligned with the wavelength of the transmitted optical signal is employed at the receiver.

**Figure 2:**
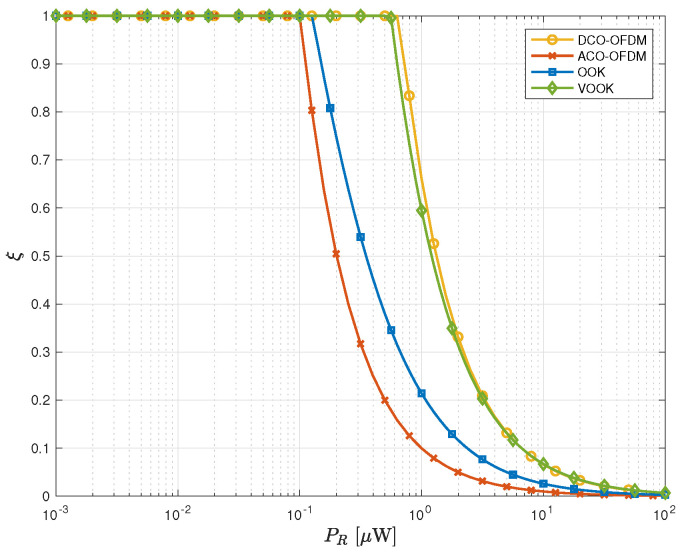
The VOA transmittance (dimensionless), ξ, versus the received signal power with background power $P_{b}=1$ nW and a dimming level γ=20%.

The adaptive change of the VOA transmittance with the change of the received optical power is the key feature of the proposed adaptive SPAD receiver. The relationship between the VOA transmittance, ξ, and the received optical signal power is shown in Figure 2 for the considered systems at γ=20% and $P_{b}=1$ nW. A similar figure or a look-up table can be implemented to cover any desired dimming level and certain background power so that in practical implementation when the environment changes the optimal VOA transmittance can be quickly determined for the proposed receiver. The transmittance of the VOA is limited up to 1, and it can be seen how this transmittance will decay as the optical power increases to keep the SPAD array in optimal performance.

### 5.1. BER Performance

**Figure 3:**
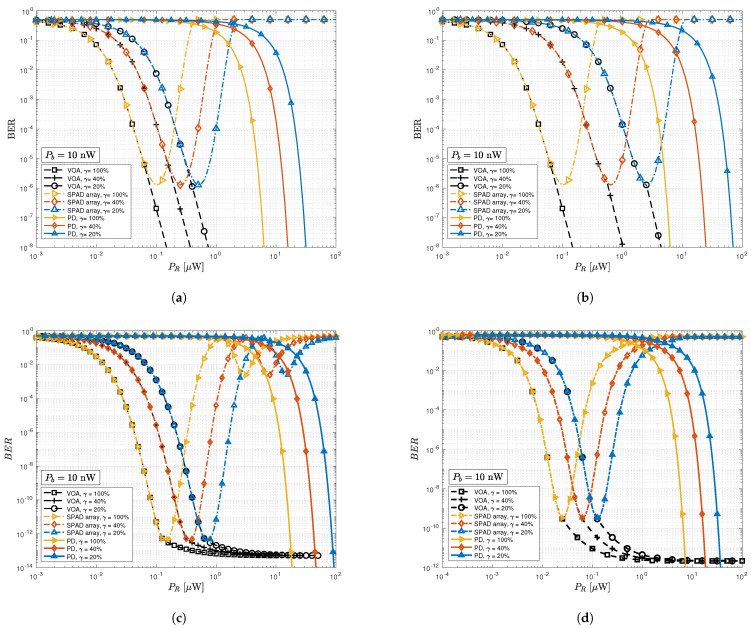
The BER versus the received signal power with background power. $P_{b}=10$ nW with different dimming levels (γ) for considered systems; (**a**) OOK; (**b**) VOOK; (**c**) 4-QAM DCO-OFDM; (**d**) 16-QAM ACO-OFDM.

The BER plots of OOK-, VOOK-, DCO-OFDM-, and ACO-OFDM-modulated signals are shown in Figure 3a–d for three dimming levels versus the received signal power, considering the higher background light power of $P_{b}=10$ nW. The sensitivity of the SPAD detector (dash-dotted curves) is significantly higher than that of the PIN PD detector (solid curves). However, as $P_{R}$ increases (e.g., due to the user’s location/orientation towards the LED), the BER of the original SPAD array receiver initially decreases and then increases due to dead-time-induced non-linear distortion caused by SPAD’s saturation. This indicates the advantage of using VOA in the proposed adaptive receiver. All the considered modulation techniques exhibit improved overall performance and effectively mitigate non-linear distortion when VOA implementation with optimal transmittance (dashed curves) is used. For instance, for γ=20% and $P_{R}=0.5\;\mu$W, the traditional OOK SPAD array achieves a BER of $10^{-6}$, however, our VOA implementation lets the SPAD array to perform at a BER = $10^{-7}$. The VOA receiver for VOOK in Figure 3b shows a similar trend, but compared with OOK, it achieves a BER = $10^{-7}$ at $P_{R}=3\;\mu$W. This also shows how VOOK is not power efficient at such dimming levels.

A comparison between DCO-OFDM and ACO-OFDM SPAD array can be seen in Figure 3c,d. For the traditional SPAD array, DCO-OFDM outperforms the ACO-OFDM in a high-power regime. For example, when $P_{R}=0.6\;\mu$W and γ=20%, SPAD receiver with DCO-OFDM can achieve a BER of $6\times 10^{-13}$. However, in such conditions, the receiver with ACO-OFDM is already in the nonlinear saturated domain with a BER of $7\times 10^{-3}$. In the low power regime, ACO-OFDM is in turn preferable. For instance, for γ=20% and $P_{R}=0.1\;\mu$W, ACO-OFDM gains a BER of $10^{-9}$ whereas the corresponding BER for DCO-OFDM is $10^{-3}$. It is also demonstrated in these figures that employing the proposed adaptive receiver can avoid the increase of BER in high power regime and continue to operate optimally.

**Figure 4:**
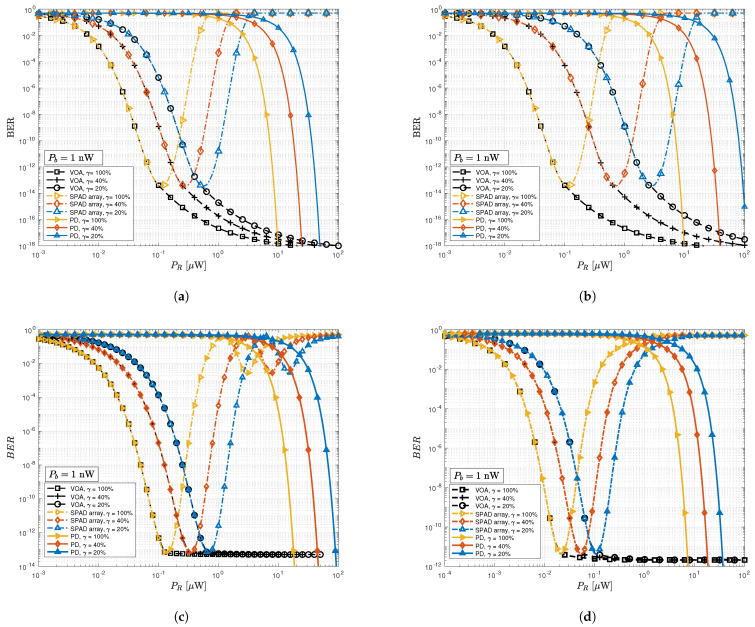
The BER versus the received signal power with background power. $P_{b}=1$ nW with different dimming levels (γ) for considered systems; (**a**) OOK; (**b**) VOOK; (**c**) 4-QAM DCO-OFDM; (**d**) 16-QAM ACO-OFDM.

When the background light intensity reduces to $P_{b}=1$ nW, indicating low ambient light conditions such as during the night, the performance of the studied systems improves as expected. As shown in Figure 4a–d, the traditional SPAD array receiver does not exhibit distortion for lower received power, matching the performance of our proposed receiver, although it starts to saturate for received optical power beyond 10 μW. It can also be concluded that increasing the dimming level leads to a degradation in the performance of the PDs.

### 5.2. Spectral Efficiency

**Figure 5:**
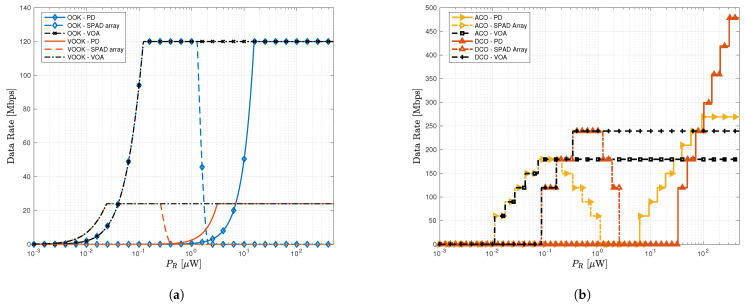
The achievable data rate versus the received signal power with a background power $P_{b}=10$ nW for the considered modulation techniques and maximum bandwidth $B_{\max}=120$ MHz for considered systems with dimming level γ=20%; (**a**) OOK vs. VOOK; (**b**) DCO-OFDM vs. ACO-OFDM.

Figure 5a,b plots the achievable data rate versus the received signal power $P_{R}$ for a dimming factor γ=20%, background power $P_{b}=10$ nW and $P_{e}^{\mathrm{th}}=3\times 10^{-3}$ BER target for the studied modulation methods. It should be noted that a vector of QAM modulation order [4,8,16,32,64] is used to attain the O-OFDM data rate results. The BERs for each $P_{R}$ when different modulation schemes are used are computed using (10), (15), (17), (24), and (55), and the maximum spectral efficiency with a BER smaller than $P_{e}^{\mathrm{th}}$ is noted.

Figure 5a shows that OOK caps at 120 Mbps as a result of the available limited 120 MHz bandwidth. It reaches this speed at $P_{R}=0.12$μW which is a considerably higher data rate than the VOOK digital dimming that reaches 24 Mbps maximum at around 24 nW when both employ VOA. This result can be explained by focusing on the codewords in Table 1, where the VOOK data bits cover only 20% of the whole codeword resulting in less background power collection compared to the OOK scheme where the pulse covers the whole symbol period. Figure 5b demonstrates that for SPAD-based receiver, DCO-OFDM can achieve a peak data rate of 239.34 Mbps when $P_{R}=0.33$μW, while for ACO-OFDM, the data rate is capped at 179.5 Mbps, which is achieved when $P_{R}=73$ nW. For both OOK and OFDM modulations, we can view that for traditional SPAD receivers, with the increase of the received optical power, the data rate first increases and then decreases as a result of non-linear distortion induced by SPAD saturation. However, as depicted in Figure 5a,b, our proposed adaptive receiver can effectively mitigate this saturation effect by monitoring the channel and adaptively changing the VOA transmittance, hence facilitating these systems to reach optimal performance and keep the performance when the received power further increases.

One can also note that at a low received optical power regime, ACO-OFDM is performing significantly better than its DCO-OFDM counterpart for all considered receivers. For example, for the SPAD receiver, when $P_{R}=10$ nW, DCO-OFDM cannot operate at this power regime, whereas the ACO-OFDM can achieve a data rate of 55.4 Mbps. The reason behind this is that ACO-OFDM in general has better power efficiency compared with DCO-OFDM. Hence, it can be concluded that ACO-OFDM is well suited to some practical applications that require a critical level of dimming control.

## 6. Conclusions

In this paper, we propose a novel adaptive SPAD-based receiver for indoor dimmable VLC systems which effectively improves the receiver sensitivity while preventing the saturation effects of SPAD detectors, resulting in decent performance over a wide range of received power and dimming levels. The use of a VOA allows the receiver to adaptively alter the optical power level incident on the SPAD array based on the received signal and background light power to achieve optimum performance. We studied the performance of the systems in the presence of single-carrier modulation schemes, such as OOK and VOOK, and multi-carrier modulation schemes, such as DCO- and ACO-OFDM. Through extensive numerical results, it is demonstrated that our proposed receiver significantly outperforms traditional SPAD and PD receivers in terms of BER and achievable data rate which validates the effectiveness of the proposed receiver in improving the performance of indoor dimmable VLC systems. In particular, depending on its application, OOK analogue dimming allows the proposed receiver to achieve a higher data rate at low dimming levels compared to the VOOK scheme, while VOOK digital dimming improves the power efficiency of the proposed receiver. Multi-carrier modulation schemes such as DCO- and ACO-OFDM support higher data rates due to their high spectral efficiency but require larger SPAD array sizes and shorter dead times. It is presented that ACO-OFDM performs better at low-light regimes and DCO-OFDM is preferable at high-light regimes.

## Figures and Tables

**Table 1:** VOOK codewords.

γ (%)	Codeword
100	dddddddddd
80	dddddddd00
60	dddddd0000
40	dddd000000
20	dd00000000
0	0000000000

**Table 2:** System parameter setting.

Symbol	Definition	Value
$\lambda_{\mathrm{op}}$	Optical wavelength	450 nm
$P_{B}$	Background light power	1 nW and 10 nW
$P_{\max}$	Maximal LED power	1 mW
$P_{\min}$	Minimal LED power	0 mW
$B_{\max}$	LED bandwidth	120 MHz
*K*	FFT and IFFT size	1024
*ℜ*	Photo-diode responsivity	0.25 A/W
$T^{o}$	Load-resistor temperature	297 K
$R_{L}$	Load resistance	50Ω

## Data Availability

Not applicable.

## References

[1] Ghassemlooy Z., Arnon S., Uysal M., Xu Z., Cheng J. (2015). Emerging Optical Wireless Communications-Advances and Challenges. IEEE J. Sel. Areas Commun..

[2] Elayan H., Amin O., Shihada B., Shubair R.M., Alouini M.S. (2020). Terahertz Band: The Last Piece of RF Spectrum Puzzle for Communication Systems. IEEE Open J. Commun. Soc..

[3] Minotto A., Haigh P.A., Łukasiewicz Ł.G., Lunedei E., Gryko D.T., Darwazeh I., Cacialli F. (2020). Visible light communication with efficient far-red/near-infrared polymer light-emitting diodes. Light Sci. Appl..

[4] Haas H., Yin L., Wang Y., Chen C. (2016). What is LiFi?. J. Light. Technol..

[5] Yoo J.H., Lee R., Oh J.K., Seo H.W., Kim J.Y., Kim H.C., Jung S.Y. Demonstration of vehicular visible light communication based on LED headlamp. Proceedings of the 2013 Fifth International Conference on Ubiquitous and Future Networks (ICUFN).

[6] Yang H., Zhong W.D., Chen C., Alphones A., Du P. (2020). QoS-Driven Optimized Design-Based Integrated Visible Light Communication and Positioning for Indoor IoT Networks. IEEE Internet Things J..

[7] Shakil Sejan M.A., Chung W.Y. (2021). Indoor Fine Particulate Matter Monitoring in a Large Area Using Bidirectional Multihop VLC. IEEE Internet Things J..

[8] Zhou H., Zhang M., Ren X. (2023). Design and Implementation of Wireless Optical Access System for VLC-IoT Networks. J. Light. Technol..

[9] Amran N.A., Soltani M.D., Yaghoobi M., Safari M. (2022). Learning Indoor Environment for Effective LiFi Communications: Signal Detection and Resource Allocation. IEEE Access.

[10] Li Y., Safari M., Henderson R., Haas H. Nonlinear Distortion in SPAD-Based Optical OFDM Systems. Proceedings of the 2015 IEEE Globecom Workshops (GC Wkshps).

[11] Huang S., Safari M. (2022). SPAD-Based Optical Wireless Communication With Signal Pre-Distortion and Noise Normalization. IEEE Trans. Commun..

[12] Fisher E., Underwood I., Henderson R. (2013). A Reconfigurable Single-Photon-Counting Integrating Receiver for Optical Communications. IEEE J.-Solid-State Circuits.

[13] Hadfield R. (2009). Single-photon detectors for optical quantum information applications. Nat. Photonics.

[14] Niclass C., Soga M., Matsubara H., Ogawa M., Kagami M. (2014). A 0.18-μm CMOS SoC for a 100-m-Range 10-Frame/s 200 ×96-Pixel Time-of-Flight Depth Sensor. IEEE J.-Solid-State Circuits.

[15] Niclass C., Rochas A., Besse P.A., Charbon E. (2005). Design and characterization of a CMOS 3-D image sensor based on single photon avalanche diodes. IEEE J.-Solid-State Circuits.

[16] Tosi A., Calandri N., Sanzaro M., Acerbi F. (2014). Low-Noise, Low-Jitter, High Detection Efficiency InGaAs/InP Single-Photon Avalanche Diode. IEEE J. Sel. Top. Quantum Electron..

[17] Chick S., Coath R., Sellahewa R., Turchetta R., Leitner T., Fenigstein A. (2014). Dead Time Compensation in CMOS Single Photon Avalanche Diodes With Active Quenching and External Reset. IEEE Trans. Electron. Devices.

[18] Bronzi D., Tisa S., Villa F., Bellisai S., Tosi A., Zappa F. (2013). Fast Sensing and Quenching of CMOS SPADs for Minimal Afterpulsing Effects. IEEE Photonics Technol. Lett..

[19] Wayne M.A., Bienfang J.C., Migdall A.L. (2021). Low-noise photon counting above 100 × 106 counts per second with a high-efficiency reach-through single-photon avalanche diode system. Appl. Phys. Lett..

[20] Xu H., Pancheri L., Braga L.H.C., Betta G.F.D., Stoppa D. (2016). Cross-talk characterization of dense single-photon avalanche diode arrays in CMOS 150-nm technology. Opt. Eng..

[21] Lee K. (2011). Modulations for Visible Light Communications With Dimming Control. IEEE Photonics Technol. Lett..

[22] Noshad M., Brandt-Pearce M. (2014). Application of Expurgated PPM to Indoor Visible Light Communications—Part I: Single-User Systems. J. Light. Technol..

[23] Carruthers J., Kahn J. (1996). Multiple-subcarrier modulation for nondirected wireless infrared communication. IEEE J. Sel. Areas Commun..

[24] Armstrong J., Lowery A. (2006). Power efficient optical OFDM. Electron. Lett..

[25] Lee S.C.J., Randel S., Breyer F., Koonen A.M.J. (2009). PAM-DMT for Intensity-Modulated and Direct-Detection Optical Communication Systems. IEEE Photonics Technol. Lett..

[26] Elgala H., Little T.D.C. (2013). Reverse polarity optical-OFDM (RPO-OFDM): Dimming compatible OFDM for gigabit VLC links. Opt. Express.

[27] Yang Y., Zeng Z., Cheng J., Guo C. (2016). An Enhanced DCO-OFDM Scheme for Dimming Control in Visible Light Communication Systems. IEEE Photonics J..

[28] Bai R., Wang Q., Wang Z. (2017). Asymmetrically Clipped Absolute Value Optical OFDM for Intensity-Modulated Direct-Detection Systems. J. Light. Technol..

[29] Shafique T., Amin O., Abdallah M., Ansari I.S., Alouini M.S., Qaraqe K. (2017). Performance Analysis of Single-Photon Avalanche Diode Underwater VLC System Using ARQ. IEEE Photonics J..

[30] Anisimova E., Nikulov D., Hu S., Bourgon M., Neumann S., Ursin R., Jennewein T., Makarov V. (2021). A low-noise single-photon detector for long-distance free-space quantum communication. EPJ Quantum Technol..

[31] Matthews W., Ahmed Z., Ali W., Collins S. (2021). A 3.45 Gigabits/s SiPM-Based OOK VLC Receiver. IEEE Photonics Technol. Lett..

[32] Huang S., Safari M. (2020). Hybrid SPAD/PD Receiver for Reliable Free-Space Optical Communication. IEEE Open J. Commun. Soc..

[33] Zafar F., Karunatilaka D., Parthiban R. (2015). Dimming schemes for visible light communication: The state of research. IEEE Wirel. Commun..

[34] Dimitrov S., Sinanovic S., Haas H. (2012). Clipping Noise in OFDM-Based Optical Wireless Communication Systems. IEEE Trans. Commun..

[35] Tsonev D., Sinanovic S., Haas H. (2013). Complete Modeling of Nonlinear Distortion in OFDM-Based Optical Wireless Communication. J. Light. Technol..

[36] Cova S., Ghioni M., Lacaita A., Samori C., Zappa F. (1996). Avalanche photodiodes and quenching circuits for single-photon detection. Appl. Opt..

[37] Eisele A., Henderson R., Schmidtke B., Funk T., Grant L., Richardson J., Freude W. 185 MHz Count Rate, 139 dB Dynamic Range Single-Photon Avalanche Diode with Active Quenching Circuit in 130 nm CMOS Technology. Proceedings of the International Image Sensor Workshop (IISW’11).

[38] Khalighi M.A., Akhouayri H., Hranilovic S. (2020). Silicon-Photomultiplier-Based Underwater Wireless Optical Communication Using Pulse-Amplitude Modulation. IEEE J. Ocean. Eng..

[39] Omote K. (1990). Dead-time effects in photon counting distributions. Nucl. Instrum. Methods Phys. Res. Sect. A Accel. Spectrom. Detect. Assoc. Equip..

[40] Yu D.F., Fessler J.A. (2000). Mean and variance of single photon counting with deadtime. Phys. Med. Biol..

[41] Kahn J., Barry J. (1997). Wireless infrared communications. Proc. IEEE.

[42] Sarbazi E., Safari M., Haas H. Photon detection characteristics and error performance of SPAD array optical receivers. Proceedings of the 2015 4th International Workshop on Optical Wireless Communications (IWOW).

[43] Hijazi M., Huang S., Safari M. Adaptive SPAD-based Receiver for Dimmable Visible Light Communication. Proceedings of the 2022 IEEE Wireless Communications and Networking Conference (WCNC).

[44] Dimitrov S., Haas H. (2013). Information Rate of OFDM-Based Optical Wireless Communication Systems With Nonlinear Distortion. J. Light. Technol..

[45] Bussgang J.J., Bussgang J.J. (1952). Crosscorrelation Functions of Amplitude-Distorted Gaussian Signals.

[46] Randel S., Breyer F., Lee S.C.J., Walewski J.W. (2010). Advanced Modulation Schemes for Short-Range Optical Communications. IEEE J. Sel. Top. Quantum Electron..

[47] Huang S., Li Y., Chen C., Soltani M.D., Henderson R., Safari M., Haas H. (2022). Performance Analysis of SPAD-Based Optical Wireless Communication with OFDM. J. Opt. Commun. Netw..

[48] Huang S., Chen C., Soltani M.D., Henderson R., Haas H., Safari M. (2022). SPAD-Based Optical Wireless Communication with ACO-OFDM. arXiv.

[49] Safari M. Efficient optical wireless communication in the presence of signal-dependent noise. Proceedings of the 2015 IEEE International Conference on Communication Workshop (ICCW).

[50] Li J., Zhang X.D., Gao Q., Luo Y., Gu D. Exact BEP Analysis for Coherent M-ary PAM and QAM over AWGN and Rayleigh Fading Channels. Proceedings of the VTC Spring 2008—IEEE Vehicular Technology Conference.

[51] Alonso O., Franch N., Canals J., Arias-Alpízar K., de la Serna E., Baldrich E., Diéguez A. (2020). An internet of things-based intensity and time-resolved fluorescence reader for point-of-care testing. Biosens. Bioelectron..

[52] Zhang L., Chitnis D., Chun H., Rajbhandari S., Faulkner G., O’Brien D., Collins S. (2018). A Comparison of APD- and SPAD-Based Receivers for Visible Light Communications. J. Light. Technol..

